# The use of implantable cardioverter defibrillators in pediatric patients

**DOI:** 10.1186/1749-8090-8-S1-O84

**Published:** 2013-09-11

**Authors:** V Campagnucci, A Silva, W Pereira, A Peroni, E Chamlian, S Gandra, A Scatolini, U Lourenço, F Silva, L Rivetti

**Affiliations:** 1 Heart Surgery, Faculty of Medical Sciences, Santa Casa de São Paulo, São Paulo, Brazil

## Background

The implant of devices in children is always a challenge. Implantable Cardioverter Defibrillator (ICD) therapy has been indicated to 14 children from March 2003 to June 2012 at Santa Casa de Sao Paulo Hospital. Ages ranged between 8 and 16 year old. The objective of this study was to analyze all pathologies, techniques, medical treatment and events related to these children.

The following diagnoses were observed: Long QT syndrome (n=2), Hypertrophic cardiomyopathy (n=3), Brugada syndrome (n=1), LV Noncompaction (n=1), Congenital Heart Disease – postoperative (n=3), Dilated Cardiomyopathy (n=1), Catecholaminergic ventricular tachycardia (n=1), Rhythmogenic right ventricular tachycardia (n=1) and Idiopathic VT (n=1). Syncope (n=4), Ventricular Tachycardia (n=6), or recovery from sudden death (n=4) was the indication for ICD.

## Methods

In all cases an endovascular endocardial approach for implanting ICDs was used. The prostheses were located below the left pectoralis major muscle, in 12 patients and below the rectus abdominis muscle in two patients. The defibrillation threshold has been distributed as follows: 15J (n=1), 20J (n=11), and 36J (n=2).

## Results

The children have been followed from one month to nine years. Each patient received pharmacological treatment for the arrhythmias with specific drugs. Seven patients had no events. Inappropriate shocks occurred in six patients. Three of them needed ablation due to atrial tachycardia. One patient had appropriate shocks. Two patients had lead dysfunction and needed replacement.

## Conclusion

cardioverter defibrillator implantation was successfully done by endovascular approach in our pediatric patients. The follow-up of this group has showed that ICDs are the solution for those children with tachyarrhythmia whose medical treatment failed.

